# Efficacy and safety of Samtropin™ recombinant human growth hormone; a double-blind randomized clinical trial

**DOI:** 10.1186/s40200-014-0115-0

**Published:** 2014-12-31

**Authors:** Ozra Tabatabaei-Malazy, Mohammad Reza Mohajeri-Tehrani, Ramin Heshmat, Eghbal Taheri, Gita Shafiee, Maryam Razzaghy-Azar, Ali Rabbani, Mostafa Qorbani, Hossein Adibi, Samimeh Shahbazi, Farzaneh Karimi, Sheema Rezaian, Bagher Larijani

**Affiliations:** Endocrinology & Metabolism Research Center, Endocrinology and Metabolism Clinical Sciences Institute, Tehran University of Medical Sciences, Tehran, Iran; Diabetes Research Center, Endocrinology and Metabolism Clinical Sciences Institute, Tehran University of Medical Sciences, Tehran, Iran; Chronic Diseases Research Center, Endocrinology and Metabolism Population Sciences Institute, Tehran University of Medical Sciences, Tehran, Iran; Inborn Error of Metabolism Research Center, Endocrinology and Metabolism Research Institute, Tehran University of Medical Sciences, Tehran, Iran; Growth & Development Center, Tehran University of Medical Sciences, Tehran, Iran; Children’s Medical Center, Tehran University of Medical Sciences, Tehran, Iran; Department of Public Health, Alborz University of Medical Sciences, Karaj, Iran; Non-Communicable Diseases Research Center, Endocrinology and Metabolism Population Sciences Institute, Tehran University of Medical Sciences, Tehran, Iran

**Keywords:** Growth hormone deficiency, Recombinant human growth hormone, Insulin like growth factor-1, Children

## Abstract

**Background:**

Recombinant human growth hormone (rhGH) can increase the growth rate in growth hormone deficient children (GHD). In this randomized clinical trial, we compared the efficacy and side effects of an Iranian brand; Samtropin with Norditropin.

**Methods:**

The GHD children were randomly treated either with standard dose of Samtropin or Norditropin rhGH for one year. Upstanding height, height standard deviation score (HSDS), growth velocity (GV), serum levels of insulin like growth factor-1 (IGF-1), and bone age (BA) were determined before and during one year treatment concomitant side effects of treatment.

**Results:**

We evaluated 22 subjects; 12 on Samtropin and, 10 on Norditropin. In each group, mean age was 12 yr and 50% of them were male. The mean differences in height, HSDS, IGF-1 and BA by Norditropin before and after 12 months were 8.8 cm, 0.5, 49 ng/ml and 2.8 yr, respectively. These measures by Samtropin were 9.1 cm, 0.6, 133 ng/ml, and 1.7 yr, respectively without any significant difference. The mean of GV by Samtropin was 9.1 vs. 8.8 cm by Norditropin without significant difference. Since the efficacy of Samtropin was found to be similar to Norditropin after 12 months; we switched to use only Samtropin for the next 12 months. The mean differences in height, HSDS, GV and BA in 20 children between months 12 and 24 were 7.0 cm, 1.6, 2.1 cm/yr and 1.0 yr, respectively (P < 0.001). We also found a non-significant decrease in IGF-1 levels. No side effects were observed.

**Conclusions:**

We need to conduct a post marketing surveillance with a large sample size in order to confirm our findings.

**Trial registration:**

Registration code number in the Iranian Registry of Clinical Trials (IRCT): IRCT1138901181414N11.

## Introduction

Recombinant human growth hormone (rhGH) has been established as an appropriate treatment to increase the growth rate in children with growth hormone deficiency (GHD), Turner syndrome (TS), Prader-Willi syndrome (PWS), chronic renal failure (CRF), and being born small for gestational age (SGA) [1]. Considering the large heterogeneity in diagnosis of GHD in children, its prevalence and incidence is largely varied in different countries [2,3]. However, its appropriate treatment is important, because these children are at higher risk of social isolation [4], anxiety and poor school performance [5], cardiovascular morbidity [6], type 2 diabetes mellitus and metabolic syndrome [7,8].

It is shown that rhGH could improve the linear growth partly through stimulation of the production of insulin like growth factor-1 (IGF-1) [9]. IGF-1 has insulin like effects, could inhibit lipolysis and activate bone remodeling [9].

The rhGH is derived from recombinant DNA and is produced by several pharmaceutical companies such as Genentech, NOVO Nordisk, Serono, etc. [10]. All of these products have equivalent therapeutic efficacy and pharmacokinetic properties [11]. Since the cost of GH therapy is substantial; it considers as an important issue for medical resources allocation. Given the current treatment costs in the United States, this correspondence to more than 50,000 $ USD per inch (2.54 cm) gained in adult height; which is a significant expense for any health system [12]. To lower the cost, one solution would be the production of rhGH by several pharmaceutical companies, especially domestic companies. Recently, Samtropin rhGH has been produced by an Iranian Pharmaceutical Company; Samen. However to establish its efficacy and safety, we should compare it with a Food and Drug Administration (FDA) approved rhGH. The aim of the present study was to demonstrate the safety and efficacy of Samtropin in comparison with Norditropin; a product that is manufactured by NOVO Nordisk Company to treat the linear growth failure in pediatric GHD. By performing this study we may be able to present the Samtropin rhGH as a less expensive drug for treatment of GHD children.

## Materials & methods

### Study population

This parallel non-inferiority/equivalence randomized clinical trial (RCT) study was carried out from August 2006 to April 2011 to compare the efficacy and side effects of Iranian brand of rhGH; Samtropin (Samen Company) to Norditropin (NOVO Nordisk Company) in pediatric GHD. Before starting the study, informed consent was obtained from all eligible children. GHD in children was diagnosed by failure to reach serum GH levels ≥10 ng/ml by clonidine GH-stimulation test] [13]. Inclusion criteria of the study was defined as non-pubertal children who have height standard deviation score (HSDS) < −3SD, or short stature children who have concomitant level of IGF-1 below that of children of the same chronological age and sex recorded for IGF-1 kit. Exclusion criteria were set as: other causes of GHD such as hypothyroidism, celiac, any acute or systemic infection diseases, seizure, HIV disorder, any chronic systemic disorders such as diabetes mellitus or CRF, TS, sleep apnea syndrome, active cancers, acute phase of craniopharengioma or other contraindications of GH replacement therapy, and concomitant use of corticosteroid.

This study included two phases. In the first phase; patients were divided in a random double blind manner into two groups; intervention group who received Samtropin and control group who received Norditropin. Both groups received the treatment over a period of 12 months, as a bed time subcutaneous injection at dose of 0.04 mg (0.1 IU)/Kgw/day, with maximum dose of 4 IU/day. We used same form of rhGH (vial) with similar color and package with a lable. The main researcher and patients were blinded to the interventions. The lable was a code cleared only for the third adviser. All the enrolled children were visited by a physician every month for the first 6 months, and then at 12^th^ month after starting treatment to assess response to rhGH treatment. The assessed parameters were height, HSDS, growth velocity (GV), weight standard deviation score (WSDS), bone age (BA), and serum levels of IGF-1. GV was determined according to below formula:

$$ \mathrm{G}\mathrm{V}=\frac{height\kern0.5em  at\kern0.5em  the\kern0.5em  end\kern0.5em  of\kern0.5em 12\kern0.5em  months\kern0.5em  after\kern0.5em  treatment(cm)- initial\kern0.5em  height\kern0.5em  before\kern0.5em  treatment(cm)}{chronogical\  age\  at\  the\  end\  of\ 12\  months\  after\  treatment(year)- initial\  chronogical\  age\  before\  treatment(year)} $$

Parents’ height was assessed before initiating the study to predict the height potential of children (PHP). We used the cross-sectional data provided by the National Center for Health Statistics (NCHS) from the Centers for Diseases Control (CDC) web site to estimate HSDS and WSDS [14]. Pubertal status was assessed and scored at each visit according to Marshall-Tunner staging [15]. Bone age was determined before and at the end of one year treatment.

Safety of the treatment was assessed by monitoring the adverse effects, measuring the body weight, performing the laboratory tests, and fundoscopy. Adverse effects were assessed in each visit according to a questionnaire consisting these signs; headache, edema, arthralgia, vomiting, hypoglycemia, genicomastia, lipoathrophia or lipohyperthrophia or local reaction in injection site, carpal tunnel syndrome, obesity, vertigo, seizure, recent visual deficit and muscle cramp. Laboratory tests included the routine hematology tests, blood chemistry, glucose metabolism (fasting blood sugar), and thyroid hormone; free thyroxin (FT4). Fundoscopy was performed at the baseline for all the patients and during the course of study forpatients who developed headache or signs of intracranial hypertension.

At the end of first year, we analyzed the findings and we found similarity in the therapeutic effects of these two brands; height, HSDS, GV, BA and IGF-1. Based on this finding, the second phase of the study was designed to examine the Samtropin’s therapy impact on growth parameters. To do this, Samtropin therapy was continued for the next 12 months for all the patients, and height and HSDS were measured at 18^th^, and 24^th^ month of the study. Moreover, probable side effects and puberty status were assessed at the above time points according to the same criteria of the first phase. Bone age, IGF-1, glucose metabolism and thyroid hormone level were determined at the end of the second year of the study.

The study was approved by ethical committee of Endocrinology and Metabolism Research Center (EMRC) of Endocrinology and Metabolism Clinical Sciences Institute of Tehran University of Medical Sciences (TUMS) and registered in the Iranian Registry of Clinical Trials (IRCT) as code number of IRCT1138901181414N11.

### Laboratory methods

Fasting blood samples were obtained from all subjects to evaluate below biochemical measures; CBC-diff, blood urea nitrogen (BUN), creatinine (Cr), alkaline phosphates (Alkp), alanine aminotransferase (ALT), aspartate aminotransferase (AST), fasting blood sugar (FBS), sodium (Na), potassium (K), FT4, and IGF-1. FBS and BUN were measured by enzymatic method, using Pars- Azmon kit/Iran. Cr was measured by JAFFE method (Pars- Azmon kit/Iran). Alkp, ALT, and AST were measured using photometer assay, enzymatic method. Na and K were measured by flame photometer Coring 480/US. CBC-diff was assessed by cell counter ABACUS. FT4 was measured by ELISA method using monobind kit/USA. IGF-1 was measured by RIA method using Immunotech kit/Chekslovaki, ranging 76–499 ng/ml for children aged more than 4 years.

### Statistical analysis

The normal distribution of data was evaluated by Kolmogrov-Smirnov analytic test. After determining the mean of variables, the student two-tailed *t* test was applied to compare the mean differences. Repeated measures were used to compare the trend of variables in different times. SPSS software (version 16) was used to perform the statistical analysis and p-values ≤ 0.05 were considered statistically significant.

## Results

### The first phase

**Figure 1:**
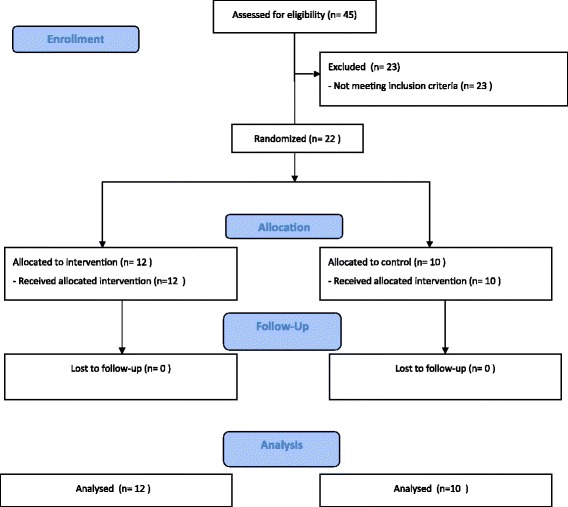
**Flow diagram of the study.**

From 45 initially evaluated subjects, after considering inclusion and exclusion criteria, 22 patients were eligible to be enrolled in the study. Flow diagram of the study is shown in Figure 1.

The baseline characteristics of 22 patients are summarized in Table 1 (10 children in NOVO group as control group, and 12 children in Samen group as intervention group). The mean age in both groups was similar (12 yr). In each group, 50% of the participants were male and most of them were in non-pubertal status. There were no significant differences in baseline characteristics of patients between two groups. All the children except one, who was undertreatment with levothyroxin, had normal FT4 levels.

**Table 1:** **Baseline characteristics of GHD children in NOVO and Samen groups**

	**NOVO group (n = 10)**	**Samen group (n = 12)**	**P value**
Chorological age (yr)	11.73 ± 1.2	12.01 ± 1.72	NS
Male/ Female (n)	5/5	6/6	NS
Pubertal status	Mixed (1 puberty)	Mixed (2 puberty)	NS
Height (cm)	129.41 ± 7.32	133.47 ± 9.91	NS
Weight (Kg)	27.40 ± 4.86	30.21 ± 7.39	NS
HSDS	−3.39 ± 0.66	−2.89 ± 0.44	NS
WSDS	−2.27 ± 0.92	−2.29 ± 1.27	NS
Bone age (yr)	8.71 ± 1.53	9.63 ± 2.26	NS
Maximum stimulated GH (ng/ml)	6.1 ± 3.7	8.0 ± 6.6	NS
IGF-1 (ng/ml)	213 ± 123	304 ± 221	NS
FBS (mg/dl)	86.10 ± 13.07	94.08 ± 3.96	NS
Cr (mg/dl)	0.62 ± 0.10	0.66 ± 0.09	NS
BUN (mg/dl)	32.00 ± 16.90	20.17 ± 10.26	NS
ALKP (mg/dl)	495.60 ± 250.82	558.92 ± 204.02	NS
ALT (mg/dl)	19.10 ± 6.26	18.82 ± 6.94	NS
AST (mg/dl)	28.40 ± 4.17	29.00 ± 6.41	NS
Na (mg/dl)	139.22 ± 1.71	139.80 ± 2.53	NS
K (mg/dl)	4.15 ± 0.35	4.13 ± 0.37	NS

Legend: HSDS: Height Standard Deviation Score, WSDS: Weight Standard Deviation Score, IGF-1: Insulin like growth factor-1, FBS: Fasting Blood Sugar, Cr:creatinine, BUN: Blood Urea Nitrogen, ALKP: Alkaline Phosphates, ALT: Alanine Aminotransferase, AST: Aspartate Aminotransferase, Na: Sodium, K: Potassium, NS: Non significant, yr: year, n: number.

Data are presented as mean ± SD according to paired *T*-Test.

**Figure 2:**
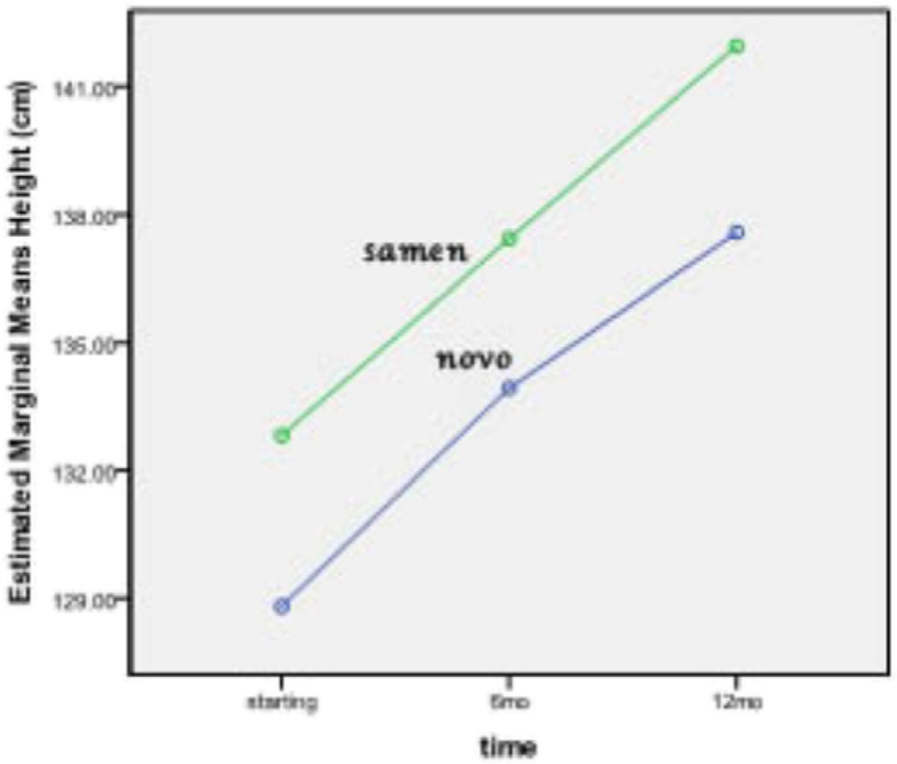
**Trend of height status in NOVO or Samen group in the first phase of the study (12 months).**

**Figure 3:**
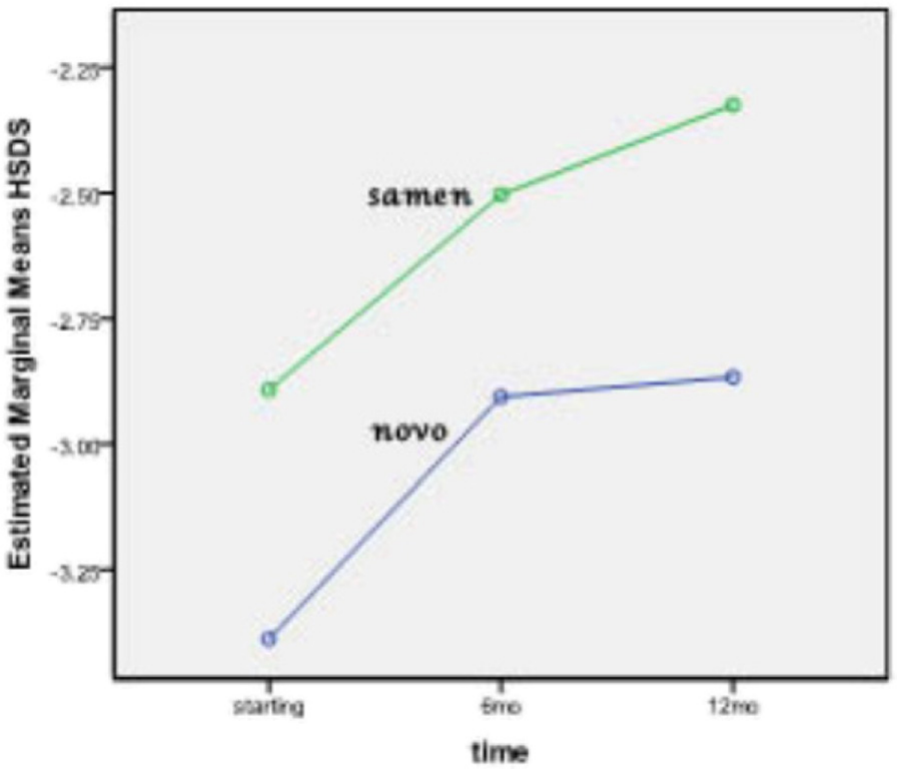
**Trend of HSDS in NOVO or Samen group in the first phase of the study (12 months).**

**Figure 4:**
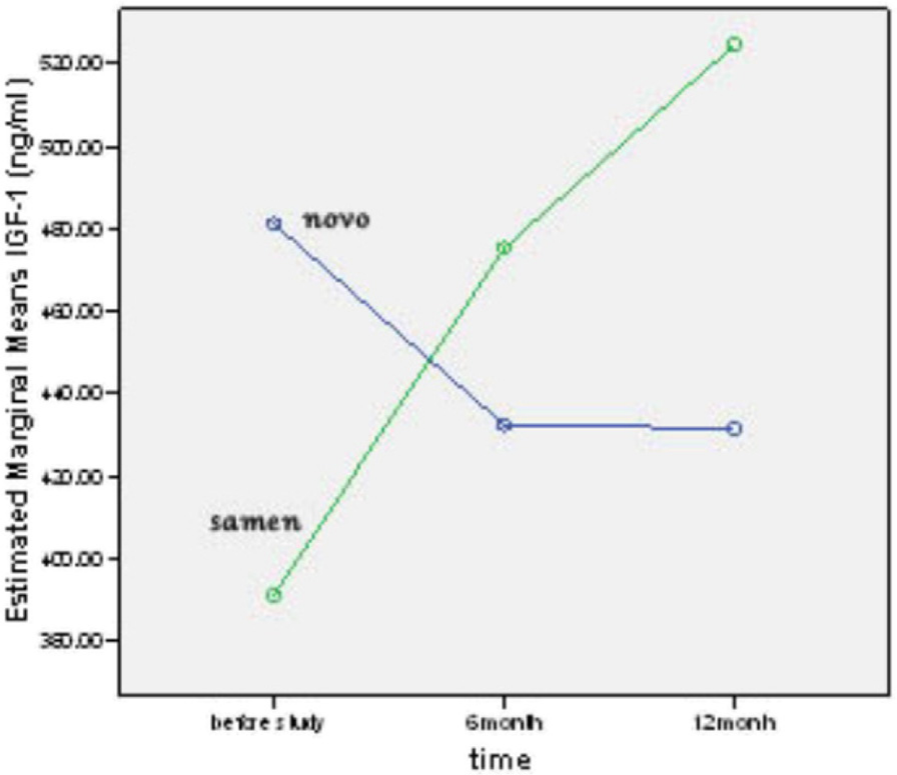
**Trend of IGF-1 means in NOVO or Samen group in the first phase of the study (12 months).**

The mean difference of height, HSDS, and IGF-1 produced by Norditropin after 12 months of treatment were 8.8 cm, 0.5, and 49 ng/ml, respectively, while these measures by Samtropin were 9.1 cm, 0.6, and 133 ng/ml, respectively. No significant difference was detected between groups (Table 2). Figures 2, 3 and 4 show the trend of change in height, HSDS and IGF-1 during 12 months treatment with NOVO or Samen rhGH.

**Table 2:** **Comparison of growth responses to Norditropin or Samtropin rhGH during 1 year treatment**

**Variables**	**Groups**	**Baseline (mean ± SD)**	**At 6** ^**th**^ **month (mean ± SD)**	**At 12** ^**th**^ **month (mean ± SD)**	**P-value of trend within group**	**P-value of trend between groups**
Height (cm)	NOVO	128.8 ± 6.5	133.9 ± 5.8	137.6 ± 6.1	<0.001*	NS
	Samen	132.8 ± 9.9	137.4 ± 9.8	141.9 ± 9.2	<0.001*	
HSDS	NOVO	−3.4 ± 0.7	−2.9 ± 0.7	−2.9 ± 0.8	0.007*	NS
	Samen	−2.9 ± 0.4	−2.5 ± 0.6	−2.3 ± 0.4	0.011*	
IGF-1(ng/ml)	NOVO	481 ± 174	432 ± 210	432 ± 199	NS	NS
	Samen	391 ± 185	475 ± 274	524 ± 263		

Legend: HSDS: Height Standard Deviation Score, IGF-1: Insulin like growth factor-1, NS:non significant.

*P ≤ 0.05 was considered statistically significant in Repeated measures Test.

The mean of WSDS in NOVO group at baseline and 12 months after start of therapy was −2.3 and −1.9, respectively, without statistically significant difference within group (p = 0.20). These figures within Samen group were −2.3 and −1.6, respectively (p = 0.009). There was no significant difference in mean difference of WSDS after 12 months rhGH therapy between groups (p = 0.22). Mean of BA from a baseline value of 8.7 year increased to 11.6 year at month 12 (P < 0.001) in NOVO group and from 9.6 year to 11.3 year (p = 0.004) in Samen group. The mean difference of BA after 1 year treatment with Norditropin rhGH was 2.8 year and with Samtropin rhGH was 1.7 (p = 0.11).

The mean of PHP for NOVO group was 159.3 cm and its difference with the baseline height of this group was 30.4 cm (P < 0.001). This difference changed to 21.7 cm at month 12 (P < 0.001). The mean of PHP in Samen group was 162.1 cm and its difference with the baseline height of this group was 29.3 cm (P < 0.001). The measure changed to a mean of 20.2 cm at month 12 (P < 0.001). No remarkable difference in mean difference height from PHP after one year treatment between two groups was detected (p = 0.74). The mean of GV induced by Samtropin rhGH was similar to Norditropin; 9.1 vs. 8.8 cm, respectively without significant difference.

Neither life-threatening adverse events nor significant changes in biochemical tests were reported during the study in both groups.

### The second phase

Since the efficacy of Samtropin rhGH was similar to Norditropin after 12 months treatment; we switched to use only Samtropin in all children for the next 12 months. Within 22 patients entered the second phase of the study, 20 children completed this phase. In this phase, we considered 12^th^ month as baseline and 24^th^ month as the end of study’s second phase. The mean of Height, HSDS and GV at the 12^th^ month of the study were 140.1 cm, −2.5 and 9.1 (Table 3). These figures changed to 147.1 cm, −0.9, and 7.0, respectively at the 24^th^ month of the study (p < 0.001).

**Table 3:** **Comparison of growth responses to Samtropin rhGH during the second phase of the study**

**Variables**	**Baseline (at 12** ^**th**^ **month) (mean ± SD)**	**At 18** ^**th**^ **month (mean ± SD)**	**At 24** ^**th**^ **month (mean ± SD)**	**P-value of Trend**
Height (cm)	140.1 ± 8.4	144.1 ± 8.0	147.1 ± 8.0	<0.001*
HSDS	−2.5 ± 0.6	−2.1 ± 0.6	−0.9 ± 0.4	<0.001*
GV(cm/yr)	9.1 ± 1.5	3.9 ± 1.6	7.0 ± 1.9	<0.001*

Legend: HSDS: Height Standard Deviation Score, GV: growth velocity.

*P ≤ 0.05 was considered statistically significant in Repeated measures Test.

**Figure 5:**
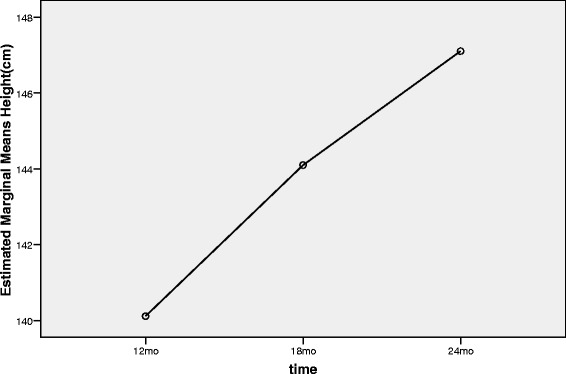
**Trend of height status in the second phase of the study (from 12** ^**th**^ **month to 24** ^**th**^ **month).**

**Figure 6:**
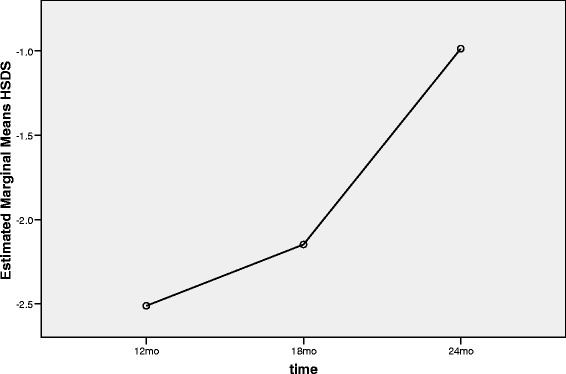
**Trend of HSDS status in the second phase of the study (from 12** ^**th**^ **month to 24** ^**th**^ **month).**

**Figure 7:**
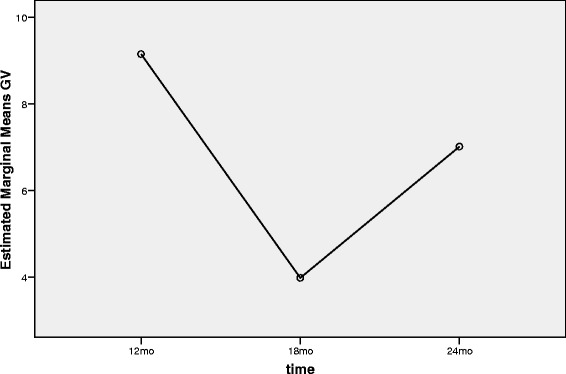
**Trend of GV in the second phase of the study (from 12** ^**th**^ **month to 24** ^**th**^ **month).**

Figures 5, 6 and 7 demonstrate the trends of height, HSDS and GV during 12 months treatment in the second phase of study.

The mean of WSDS and BA at baseline of the second phase was −1.8 ± 1.5 and 11.4 ± 1.9 yr, respectively, which was improved significantly to 0.6 ± 1.3 and 12.4 ± 2.0 yr (p < 0.001 and p < 0.05), respectively after 12 months of treatment. A non-significant decrease (p = 0.26) in serum IGF-1 levels was detected between baseline (539 ± 268 ng/ml) and month 24 of the study (429 ± 220 ng/ml). The mean difference in PHP between baseline and month 12 of the study was 20.6. This figure declines to 13.6 cm (P < 0.001) at month 24.

Similar to results reported in the first phase of the study, in the second phase we found neither life-threatening adverse events nor significant changes in laboratory data.

## Discussion

### Auxological parameters

Height, HSDS, and GV are well established measures of linear growth during rhGH treatment, particularly during the usual pre-pubertal age [16,17]. In our study we found an improvement in height, HSDS, and GV values which could be assumed as the efficient growth response of the children to Samtropin. Similar to other published studies [18-20] for all these auxological parameters, significant differences were observed between values of baseline and month 12 of the study within NOVO or Samen groups. However, no significant difference between these two groups was observed. Moreover, this treatment response was not affected by switching from Norditropin to Samtropin in the second phase of the study.

After one year treatment with Samtropin, height difference was 9.1 cm, a change that waseven more than height gained by Norditropin treatment (8.8 cm). It is generally accepted that an increase of at least 0.25 SD in HSDS over the first year of treatment is a definitive evidence of rhGH efficacy [16]. The increase in HSDS after treatment with rhGH therapy in our study was 0.57 by Samtropin and 0.52 by Norditropin without any significant difference between them. When we considered the two years treatment by rhGH in our patients regardless of the brand employed, we found the significant change in HSDS from −2.5 at baseline to −0.9, a figure that is in agreement with other studies [21-23]. Root et al. [21] reported that HSDS increased from a mean value of −3.4 to −1.5 after 4 years treatment with Omnitrope in 140 GHD children. Coste et al. [22] reported in French GHD children a HSDS of −2.0 compared with a HSDS of −1.3 after 4 years of treatment with Omnitrope. These figures changed from −2.2 at baseline to −0.9 after 4 years treatment with Omnitrope in British children [23].

The standard curves of height could be affected by the pubertal growth spurt. On the other hand, in children with delayed puberty the changes in HSDS can be masked by using rhGH. Therefore, it seems that GV value may be a more appropriate measure of rhGH response in such children. Inboth our groups, the mean GV increased equally (9.1 vs. 8.8 cm/year), after 12 months of treatment with rhGH. The mean GV increased equally again in both groups (2.1 cm/year) in the second phase of study. Similar to findings of the other published studies [19,22,24,25] the greatest acceleration in GV was occurred during the first year of the rhGH treatment in our study.

### Pharmacodynamic parameter

IGF-1 is defined as the preferred pharmacodynamic marker for assessing theactivity of rhGH in similar medicinal products containing somatropin [26]. IGF-1 levels tend to be low in GHD and rise in a dose-dependent manner in children treated with rhGH [27]. Scire et al. [28] demonstrated that at a conventional rhGH dose (0.03 mg/kg/day) abnormal elevation of IGF-1 in GHD children does not occur. In our study, Samtropin similar to Norditropin stimulated the synthesis of IGF-1 which was detected by significant increase in IGF-1 serum level after 12 months of treatment. The increase was more pronounced during the first 6 month of treatment, the time period when the growth of patients was observed to be most accelerated. The increase in IGF-1 level from baseline to which achieved after 12 months treatment with Samtropin in our study was very similar to other studies [18,29]. The mean level of IGF-1 was decreased non-significantly in the second phase of study.

### PHP and bone maturation

The PHP corresponds to the height that patient is expected to reach at the end of linear growth. In our study, the mean difference of PHP and the height of children after 1 year treatment with rhGH in NOVO group was 30.6 cm and for Samen group was 29.3 cm, without any significant difference between groups. When we continued the rhGH therapy with Samtropin for the second year, the mean difference between PHP and the height of children was 13.63 cm. It is shown that responses to rhGH could be influenced by several factors such as dosages of rhGH, degree of delay in growth and skeletal age before initial treatment, and manipulation of puberty [30]. Before starting treatment, we had 3 children who were in pubertal stage; one child in NOVO group and 2 children in Samen group. The number of pubertal kids increased to 11 subjects at the end of 2 years. This fact could lead to variation in response to rhGH particularly in the second year of the study.

After 1 year treatment with Samtropin the bone age of our patients increased by 1.71 year, whilst this figure for Norditropin was 2.85 year. Although the effect of Samtropin on bone age was lower than the effect of Norditropin, there was not any significant difference between treatment groups. The effect of Samtropin on the bone age in the second phase was equal to 1 year. The effect of Samtropin on bone age for the second year was equal to the chronological age. This finding is important since itshows the normal pace of bone maturation, and also illustrates that skeletal growth induced by Samtropin is similar to other rhGH products including Omnitrope and Saizen [18,20]. Similar to our other findings, the most pronounced effect of Samtropin was produced in the first year of treatment. In the other word, the potential gain in height decreased as the treatment period was increased.

### Safety

Mean values of blood chemistry were within normal ranges during study. Monitoring of free T4 and TSH is recommended for detecting hypothyroidism which may appear during rhGH therapy [31,32]. None of our studied children developed low FT4 while were on rhGH therapy. The rhGH therapy may induce carbohydrate intolerance in children with compromised insulin secretion [33]. None of our children in two phases of the study developed glucose intolerance asmeasured by FBS. This finding is in agreement with Aman et al. [34] and Soliman et al. [35] findings who demonstrated that rhGH therapy could not produce any negative effect on FBS. Overall the safety profile of our treatments was similar to findings of other published studies [29,35] with no significant changes between our groups. All of our safety assessment results were in agreement with other studies that reviewed the short and long term safety of rhGH therapy [36-38]. The most frequent adverse effects reported by other products were headache, eosinophilia, hypothyroidism and injection site reactions [19]. Overall the side effects of rhGH therapy are uncommon and reversible after discontinuation or dose reduction of treatment [30].

### Limitations of the study

We have to mention that our experiment has some limitations. We did not measure the free IGF-1 or IGF binding protein-3 (IGFBP-3) as a carrier of IGF-1 in serum. Serum level of IGF-1 reflects the 24-hour secretion of GH. However, the general believe is that free IGF-1 is a biologically active component of IGF-1 [29]. Furthermore, the free IGF-1 is controlled by IGFBP-3 [29]. Our small sample size is another limitation. We understood that we need to design a post marketing surveillance with a larger sample size in order to confirm our findings, however the result of this study have implications for clinical practice and health policy.

## Conclusions

In conclusion, the clinical comparability between Norditropin and Samtropin demonstrated that Samtropinat a dose of 0.04 mg/kg/day is effective to increase the linear growth, safe and well tolerated both locally and systemically within 12 months treatment of GHD children. Although, the results of this study are supportive of the long term (up to 2 years) benefits of Samtropin rhGH in GHD children, interpretation of the finding should be done with caution since the second phase of our study was not a comparative, randomized trial. Considering that only small number of our patients received Samtropin for the full 2 years, the long term (2 years) treatment with Samtropin was efficient and well tolerated.

## Abbreviations

rhGH: Recombinant human growth hormone
GHD: Growth hormone deficient
TS: Turner syndrome
PWS: Prader-Willi syndrome
CRF: Renal failure
SGA: Small for gestational age
IGF-1: Insulin like growth factor-1
FDA: Food and Drug Administration
RCT: Randomized clinical trial
HSDS: Height standard deviation score
GV: Growth velocity
WSDS: Weight standard deviation score
BA: Bone age
PHP: Predict height potential
NCHS: National Center for Health Statistics
CDC: Centers for Diseases Control
FT4: Free thyroxin
EMRC: Endocrinology and Metabolism Research Center
TUMS: Tehran University of Medical Sciences
IRCT: Iranian Registry of Clinical Trials
BUN: Blood urea nitrogen
Cr: Creatinine
Alkp: Alkaline phosphates
ALT: Alanine aminotransferase
AST: Aspartate aminotransferase
FBS: Fasting blood sugar
Na): Sodium
(K: Potassium
IGFBP-3: IGF binding protein-3
